# Distributed optical fiber sensing for metal particle defect detection in GIS via frequency-swept interferometry

**DOI:** 10.1371/journal.pone.0355959

**Published:** 2026-08-11

**Authors:** Xu Zhixin, Zhao Xincheng, Li Chaohui, Su Honghui, Xie Peng, Yang Liusong

**Affiliations:** Ultra-High Voltage Branch, State Grid Fujian Electric Power Co., Ltd., Fuzhou, China; Hohai University, CHINA

## Abstract

Conventional detection of metal particle defects in gas-insulated switchgear (GIS) mainly relies on piezoelectric (PZT) sensors, which are inadequate for distributed monitoring in modern power equipment. Although interferometric optical fiber sensors offer high sensitivity for GIS partial discharge detection, they are generally limited to single-point measurements. To address this issue, this study proposes a multiplexing method for optical fiber acoustic emission sensors based on optical frequency-scanning in a Michelson interferometric configuration. By introducing high-frequency carriers through optical frequency modulation and separating channels via bandpass filtering, the proposed method preserves the high sensitivity of conventional single-point interferometric sensors and extends it to multiplexed sensing, enabling frequency-division multiplexing of multiple sensors using a single demodulation system. Experiments on a 126 kV GIS with four sensing units demonstrate that the proposed system can effectively detect discharge signals and identify the defective cavity. This work provides a promising approach for distributed GIS condition monitoring.

## I. Introduction

Gas-insulated switchgear (GIS) is a core component of power systems, and its reliable operation is critical for the stability of the electrical grid [1,2]. However, long-term operational experience shows that insulation failure due to metal particle defects occurs frequently in GIS [3–6]. Therefore, enhancing the detection of metal particle defects in GIS is a key focus for power grid operation and maintenance [7,8].

Under operating voltage, metal particles in GIS may exhibit jumping behavior and induce partial discharge [9], enabling their detection via electrical methods such as ultrahigh-frequency (UHF) techniques. However, electrical signals are highly susceptible to electromagnetic interference [10], making it difficult for electrical methods to reliably capture effective information. Consequently, acoustic detection methods based on piezoelectric ceramic (PZT) sensors have been commonly employed. This approach can detect the acoustic emission (AE) signals generated by the movement of particles and capture the ultrasonic waves produced during discharges [11,12]. However, PZT sensors exhibit limited reusability, while the strong attenuation of acoustic signals in large-scale GIS systems creates a pressing need for synchronous multipoint detection, which is difficult to achieve with PZT-based approaches.

Recent advances in optoelectronics have made fiber-optic acoustic emission sensing a promising alternative to PZT sensors [13], with interferometric techniques attracting particular attention [14–17]. For instance, Ma et al. proposed a Michelson interferometer-based optical fiber AE sensor for metal particle detection in GIS [18,19], and further improved its sensitivity through structural optimization in 2023 [20]. Jiang et al. designed an AE sensing system based on a Sagnac interferometer structure. Their experimental results indicated good consistency between the optical fiber sensor and high-frequency current sensor. Chen et al. [21] proposed an embedded GIS partial-discharge acoustic emission detection technology based on a Michelson interferometric structure, achieving the detection of discharge defects from free metal particles at 15.3 pC. In addition, related studies have reported partial-discharge AE sensing based on various all-fiber interferometric configurations [22–24]. Collectively, these studies have established the feasibility and sensitivity advantages of interferometric fiber-optic AE sensing for partial-discharge detection. However, the primary focus of most existing studies has been the improvement of sensing sensitivity through interferometer configuration or sensing-unit structural optimization. Their sensing systems generally employ a single sensing unit with an independent interrogation and demodulation link, and therefore operate as discrete point-sensing systems. Consequently, they cannot readily provide multiplexed detection.

Unfortunately, acoustic vibration signals attenuate significantly when propagating through spacers in GIS equipment, often necessitating the deployment of multiple sensors for multipoint synchronous detection in a single experiment. Therefore, a multiplexed sensing method is needed to reduce the number of independent sensing links and improve the practical applicability of fiber-optic AE sensing in GIS condition monitoring.

Regarding multiplexed sensing, distributed fiber-optic sensing schemes based on optical frequency-domain reflectometry (OFDR) [25] and optical time-domain reflectometry (OTDR) [26] have been reported. For example, Zhang et al. [27] developed an OTDR-based monitoring system for overhead transmission-line icing, in which vibration signals were used to characterize conductor jumping during de-icing. Lisbel et al. [28] applied an OTDR-based method to monitor broken-wire defects in prestressed concrete cylinder pipes. However, these Rayleigh-backscattering-based distributed sensing techniques are generally constrained by the weak intensity of the backscattered light. In addition, the measurement rate of OTDR-based systems is limited by the round-trip propagation time of the probe signal, whereas OFDR-based systems are affected by the laser sweeping rate and signal-processing bandwidth. As a result, simultaneously achieving high sensitivity and wide dynamic bandwidth remains challenging. Therefore, these techniques are mainly suitable for large-amplitude and relatively low-frequency strain or vibration sensing. In contrast, discharge-induced acoustic-emission signals are characterized by high frequency and small amplitude, imposing more stringent requirements on the sensitivity and dynamic response of the fiber-optic sensing system.

To address this issue, this study proposes a multiplexing method for Michelson fiber-optic interferometers based on optical frequency sweeping. Unlike Rayleigh-backscattering-based distributed sensing methods, the proposed method does not rely on weak distributed backscattered signals for acoustic detection. Instead, optical-frequency sweeping is introduced into the conventional Michelson interferometric sensing structure to generate high-frequency carrier components according to the optical-path differences of different sensing branches. This design preserves the high phase sensitivity of Michelson fiber-optic interferometers for high-frequency AE signal detection, while enabling the signals from multiple sensing units to be separated by their carrier-frequency differences. Therefore, multiple fiber-optic sensors can be multiplexed and demodulated using a common interrogation system. Based on this method, the key parameters of the optical-frequency-swept interferometric sensing system are designed in this study. An experimental platform is then established to conduct partial-discharge detection experiments, and the multiplexed detection capability of the proposed sensing system for metal-particle defects in GIS is evaluated.

## II. Materials and methods

### A. Principle of acoustic emission sensing

**Fig 1:**
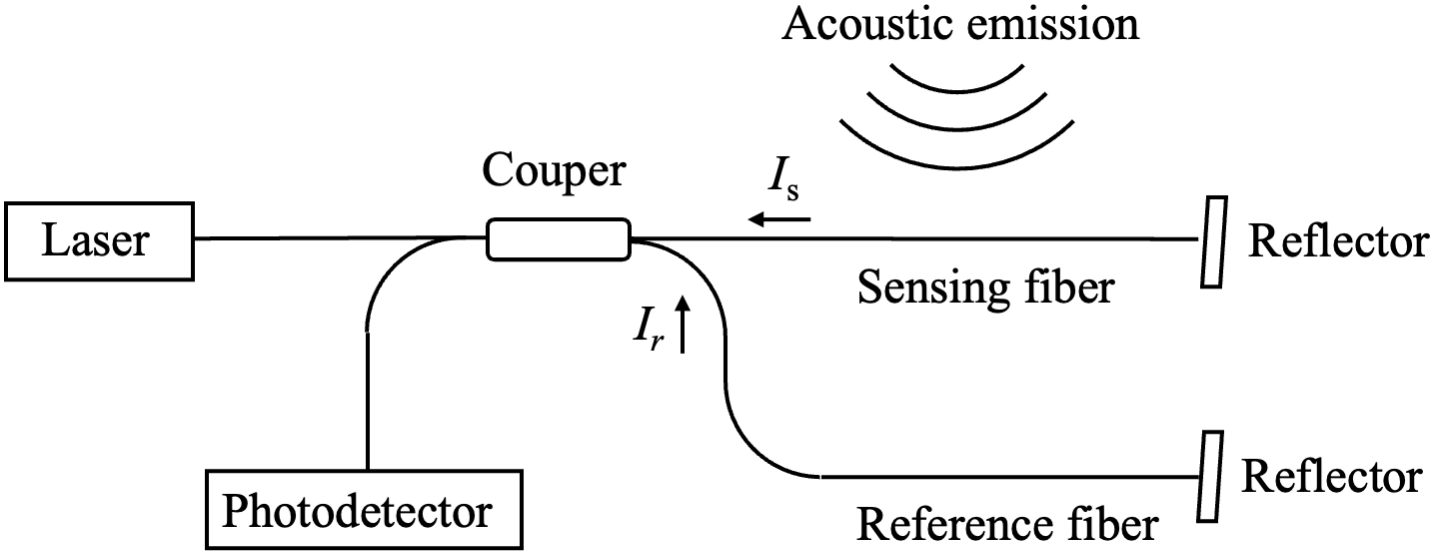
Basic structure of the Michelson interferometer-based optical fiber AE sensing system.

A Michelson interferometer-based optical fiber AE sensing system operates by detecting phase variations in the transmitted light induced by acoustic waves. The basic configuration of the sensing system is shown in Fig 1.

The light emitted by the laser is separated by a fiber coupler into sensing and reference beams, each entering its respective fiber. Both beams are reflected by the end fiber mirror and return along the same path, where interference occurs at the fiber coupler. Let the light fields of the two interfering beams in the system be denoted as *E*_s_ and *E*_r_, respectively, as shown in [25].


$$\begin{array}{c} E_{s}\left(t\right)=E_{s0}\;exp\left\{j\left[2\pi f_{\text{0}}\left(t-\tau_{s}\right)\right]\right\} \end{array}$$ (1)

$$\begin{array}{c} E_{r}\left(t\right)=E_{r0}\;exp\left\{j\left[2\pi f_{\text{0}}\left(t-\tau_{r}\right)\right]\right\} \end{array}$$ (2)

In the equation, *t* is time, *f*_0_ is the optical frequency of the laser, *E*_s0_ is the amplitude of the sensing light field, *E*_r0_ is the amplitude of the reference light field, *τ*_s_ and *τ*_r_ are the round-trip transmission times of light in the sensing and reference arms, respectively.

After interference occurs, the interference light intensity *I* can be expressed as [29]


$$\begin{array}{c} \begin{array}{c} I(t)=\frac{cn\epsilon_{\text{0}}}{\text{2}}[E_{s}\left(t)+E_{r}(t\right)]\cdot [E_{s}(t)+E_{r}(t){]}^{*}=\frac{cn\epsilon_{\text{0}}}{\text{2}}\{E_{r0}^{\text{2}}+E_{s0}^{\text{2}}+2E_{r0}E_{s0}cos\left[2\pi f_{\text{0}}\left(\tau_{s}-\tau_{r}\right)\right]\} \end{array} \end{array}$$ (3)

Where *c* is the speed of light in vacuum, *n* is the refractive index of the fiber, and *ε*_0_ is the permittivity of vacuum.

The intensity of the sensing light *I*_s0_ and reference light *I*_r0_ can be expressed as


$$\begin{array}{c} I_{s0}=\frac{cn\varepsilon_{\text{0}}}{\text{2}}E_{s0}^{\text{2}} \end{array}$$ (4)

$$\begin{array}{c} I_{r0}=\frac{cn\varepsilon_{\text{0}}}{\text{2}}E_{r0}^{\text{2}} \end{array}$$ (5)

Substituting Eqs (4) and (5) into Eq (3) obtains:


$$\begin{array}{c} I\left(t\right)=I_{s0}+I_{r0}+2\sqrt{I_{s0}I_{r0}}\text{cos}\left(2\pi f_{\text{0}}\tau \right) \end{array}$$ (6)

Where τ is the inter-beam time difference, which can also be expressed as


$$\begin{array}{c} \tau =\tau_{s}-\tau_{r}=\frac{nl}{c} \end{array}$$ (7)

Where *l* is the optical path difference between the interferometer-sensing fiber and reference fiber.

When AE signals generated by metal particle interact with the sensing fiber, they induce dimensional changes that alter the initial optical path difference 𝑙. This introduces an additional phase variation in the interference light phase, denoted as Δφ. Therefore, Eq. (6) can be expressed as:


$$\begin{array}{c} I\left(t\right)=I_{s0}+I_{r0}+2\sqrt{I_{s0}I_{r0}}cos(\varphi +\Delta \varphi ) \end{array}$$ (8)

In the equation,φ represents the initial phase, which is given by:


$$\begin{array}{c} \varphi =2\pi f_{\text{0}}nl/c \end{array}$$ (9)

Therefore, the external acoustic signal can be detected by demodulating the phase variation of the interference signal.

### B. Principle of optical sweep frequency interferometric sensing

In this experiment, the length of the reference fiber was approximately 0.5 m, corresponding to a round-trip transmission time of about 4.9 ns, which is much smaller than the round-trip delay of the sensing arm and can therefore be neglected. If the frequency of the laser output in Fig 1 begins to increase linearly at a constant rate from *f*_0_, the laser light field returning from the reference fiber to the coupler *E*_r_ can be expressed as


$$\begin{array}{c} E_{r}\left(t\right)=E_{r0}\;exp\left[j\left(2\pi f_{\text{0}}t+\pi \gamma t^{\text{2}}\right)\right] \end{array}$$ (10)

whereγ is the laser sweep rate.

Since there is an optical path difference between the sensing and reference fibers, the laser light field returning from the sensing fiber to the coupler, Es, can be expressed as


$$\begin{array}{c} E_{s}\left(t\right)=E_{s0}\;exp\left[j\left(2\pi f_{\text{0}}(t-\tau +\pi \gamma (t-\tau {)}^{\text{2}}\right)\right] \end{array}$$ (11)

Based on the previous derivation, when subjected to external acoustic signals, the intensity I of the two interfering light beams can be expressed as


$$\begin{array}{c} I\left(t\right)=I_{s0}+I_{r0}+2\sqrt{I_{s0}I_{r0}}\;cos[2\pi \gamma \tau t+\varphi_{\gamma }+\Delta \varphi ]\} \end{array}$$ (12)

Where φ represents a constant phase, which is given by:


$$\begin{array}{c} \varphi =2\pi f_{\text{0}}\tau -\pi \gamma \tau^{\text{2}} \end{array}$$ (13)

By comparing Eq. (8) and (12), it can be observed that high-frequency components can be introduced into the phase of the interference light signal using the optical frequency sweeping method. The frequency of this component is related to the initial length difference l between the sensing and reference fibers. Therefore, if there are multiple sensing fibers, each with a different optical path difference relative to the reference fiber, the interference light signal will consist of multiple superimposed frequency components.

**Fig 2:**
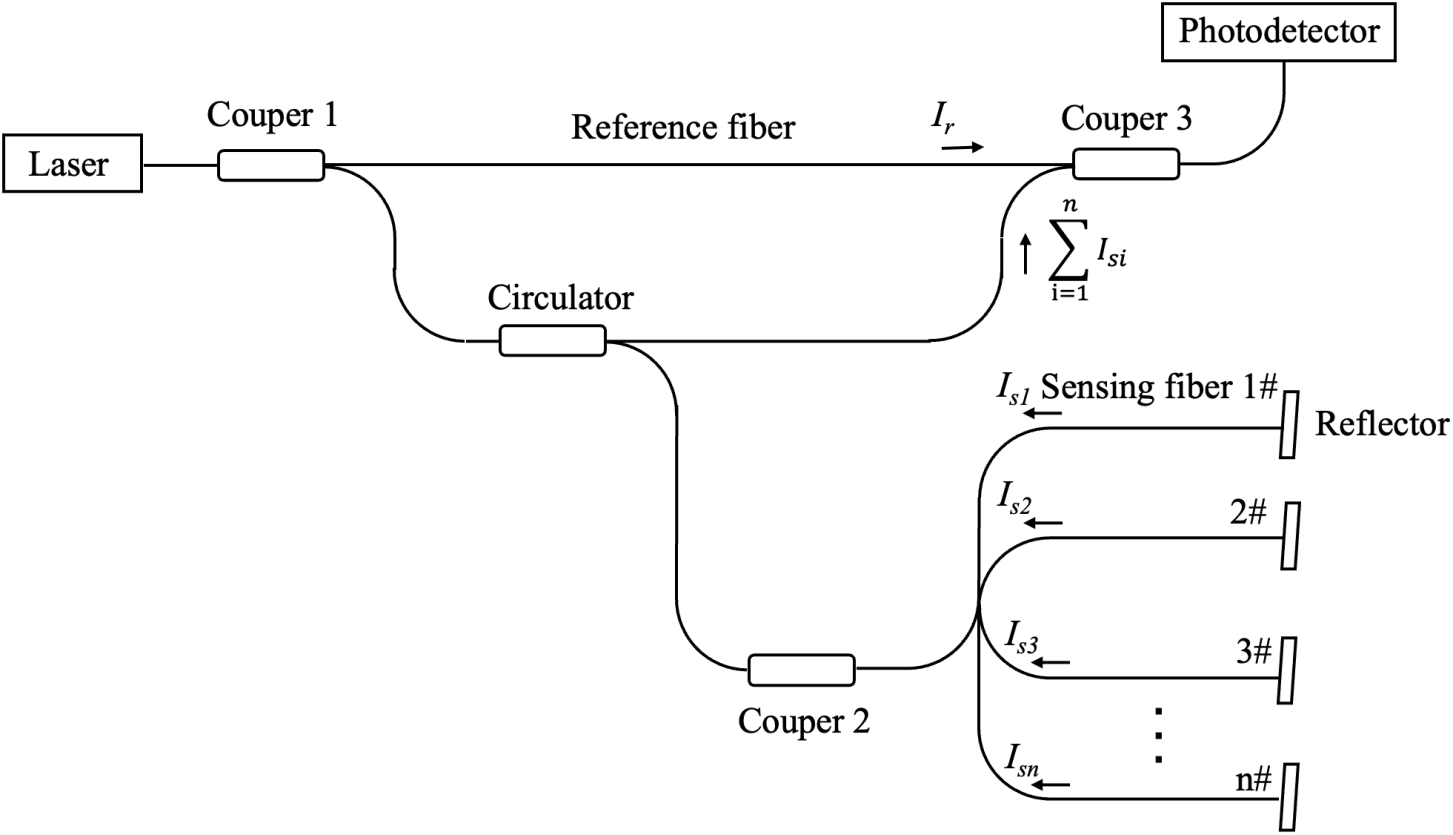
Distributed optical fiber sensing system topology.

Based on this principle, the topology of the distributed fiber-optic sensing system designed for this project is shown in Fig 2.

As shown in Fig 2, the laser output is divided into two paths after passing through Coupler 1; one path enters the reference light path, while the other enters the sensing light path. In the sensing light path, the laser passes through a fiber-optic circulator and is split into n paths by 1 × 4 Coupler 2, with each path terminating in a mirror. The reflected light returns along the original path, passes through the fiber-optic circulator, and enters Coupler 3, where interference occurs. At this point, the alternating component of the interference light intensity *I* can be expressed as


$$\begin{array}{c} I\left(t\right)=\sum_{i=1}^{n}2\sqrt{I_{s0i}I_{r0}}cos[2\pi \gamma \tau_{i}t+\varphi_{\gamma i}+\Delta \varphi_{i}]+M\left(t\right) \end{array}$$ (14)

Where *I*_s0i_ represents the amplitude of the sensing light intensity through the *i*-th sensing fiber,$\tau_{i}$ is the time difference between the *i*-th sensing light and the reference light,$\Delta \varphi_{i}$ is the constant phase corresponding to the *i*-th sensing light after interference,$\Delta \varphi_{i}$ is the change in light phase caused by external signals acting on the *i*-th sensing fiber, and M(t) is the noise term generated by the interference of the various sensing lights at Coupler 3. This noise term contains signals with multiple frequency components, with the maximum frequency depending on the difference in the round-trip transmission times of the laser in each sensing fiber. By setting appropriate lengths for the sensing fiber, this noise term can be maintained within a low-frequency range. The specific selection of the sensing-fiber lengths and the corresponding carrier-frequency allocation are quantitatively discussed in the following system-design section.

From Eq. (14), it can be seen that by setting different lengths for the sensing fiber, resulting in variations in$\tau_{\text{1}}$, the interference signal frequencies from each sensing fiber will differ. Therefore, the signals converted from optical to electrical can be separated through bandpass filtering, yielding the target signals *U*_i_ corresponding to each sensing fiber (*i* = 1–n). This allows for frequency-division multiplexing of the sensor devices. Ui can be expressed as:


$$\begin{array}{c} U_{i}\left(t\right)=U_{io}\;cos[2\pi f_{i}t+\varphi_{\gamma i}+\Delta \varphi_{i}] \end{array}$$ (15)

Where *U*_io_ represents the amplitude of the alternating component after photoelectric conversion, and *f*_i_ is the carrier frequency, which is given by


$$\begin{array}{c} f_{i}=\gamma \tau_{i}=\gamma \Delta l_{i}n/c \end{array}$$ (16)

Where Δ*l*_i_ is the length difference between the *i*-th sensing fiber and the reference fiber.

### C. Design of optical fiber sensing structure

The sensing fiber is the core component of the distributed fiber-optic sensing system. From the previous analysis, it is evident that under the same external signal, a larger change in the dimensions of the sensing fiber results in a greater phase change in the interference signal, indicating a higher system sensitivity. Therefore, it is essential to maximize the deformation of the sensing fiber under the influence of acoustic vibration signals.

**Fig 3:**
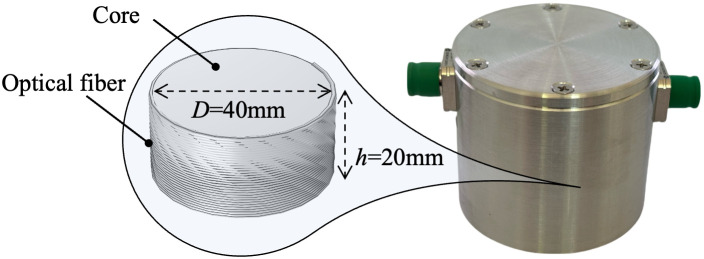
Physical diagram of the optical fiber sensing unit.

Since the main objective of this work is to investigate the optical-frequency-swept multiplexing method rather than to optimize the sensing-head structure, the sensing-unit structure was designed with reference to the high-sensitivity fiber-optic acoustic sensor reported in [30], where a fiber-wound stainless-steel-core structure was experimentally demonstrated to be effective for GIS partial-discharge acoustic-emission detection. Following this validated design, a 125 m long optical fiber was wound around a stainless-steel core with a diameter of *D* = 40 mm and a height of *h* = 20 mm to form one sensing unit. In addition, a packaging structure was designed to improve the long-term reliability of the fiber-optic sensing unit, as shown in Fig 3.

To maintain channel consistency in the multiplexed sensing system, all sensing units were fabricated using the same core dimensions, fiber length, fiber winding method, and packaging structure. The optical-path length differences required for carrier-frequency allocation were achieved by adjusting the lengths of the connecting fibers placed before the individual sensing units, rather than by changing the sensing-unit structure itself.

### D. Distributed sensing system design

**Fig 4:**
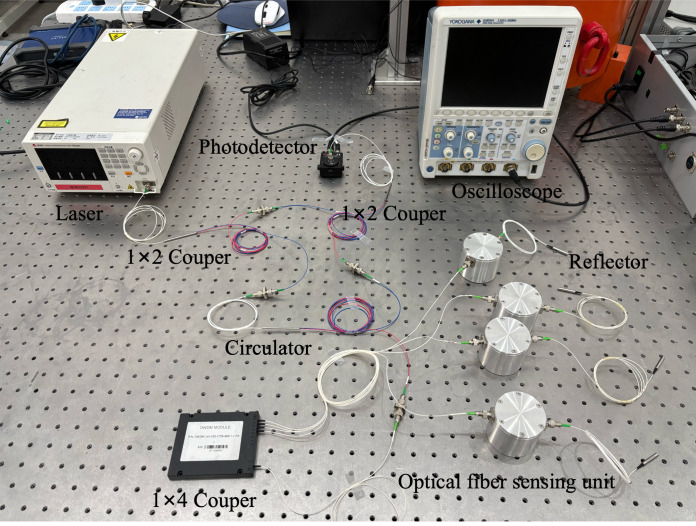
Photograph of distributed optical fiber sensing system.

In this paper, four optical fiber sensing units were used, into the topology in Fig 2, denoted as sensors 1–4. In addition, a tunable laser (Santec TSL-510) with an output optical power of 10mW was used as the light source, which can achieve fast frequency sweeping in the range of 1480–1640 nm. The output laser was divided into two paths after passing through a 1 × 2 fiber coupler, one path entered the reference fiber, and the other path passed through a fiber circulator and then through a 1 × 4 fiber coupler, and was divided into four paths, entering the aforementioned four fiber sensing units. A Faraday rotating mirror was connected to the end of each sensing unit to reflect the four sensing light beams to another 1 × 2 fiber coupler for interference. The interference light signal was converted into photoelectricity using a photodetector (Thorlabs APD130C) and then acquired using an oscilloscope (Yokogawa DLM2034). The distributed fiber sensing system is shown in Fig 4.

From the previous analysis, the keys to achieving separation and demodulation of the four sensing signals are: 1) the frequency bands of the target signals *U*_i_ (*i* = 1–4) corresponding to each sensor do not overlap, and 2) the frequencies of the noise signals M caused by the interference of the optical signals in the light paths at Coupler 3 are all less than the frequencies of the corresponding target signals *U*_i_ (*i* = 1–4).

From Eq. (12), it can be inferred that the target signals *U*_i_ (*i* = 1–4) corresponding to each sensor can be regarded as carrier signals with a carrier frequency of fi, while the changes in light phase caused by external acoustic signals can be viewed as phase modulation of these signals. According to Carson’s bandwidth theorem, the effective bandwidth EBD of the carrier signal (i.e., the target signal Ui corresponding to each sensor) can be expressed as [31]


$$\begin{array}{c} EBD=2(\varphi_{A}+1)f_{u} \end{array}$$ (17)

In this context, *f*_u_ represents the frequency of the external acoustic vibration signal. According to previous studies on GIS partial-discharge acoustic-emission detection, the dominant frequency components of discharge-induced AE signals are mainly distributed in the range of 20–80 kHz [32]. Therefore, *f*_u_ = 80 kHz was selected as the upper-bound acoustic frequency in this study. $\varphi_{A}$ denotes the amplitude of phase variation in the interference light induced by the signal under investigation. In fiber-optic interferometric sensing systems, phase-generated carrier (PGC) demodulation commonly introduces a carrier phase modulation depth at the radian level, typically around 2 rad, to obtain stable demodulation performance [33]. By contrast, discharge-induced AE signals are weak acoustic perturbations superimposed on the interferometric phase, and their phase modulation depth is generally smaller than the intentionally introduced PGC carrier modulation depth. Therefore, $\varphi_{A}$ =2 rad was adopted as a conservative upper-bound design value for the acoustic-induced phase modulation depth. Consequently, the effective bandwidth EBD is determined to be 480 kHz.

To avoid crosstalk between the multiple signals in this distributed sensing system, the carrier frequency difference between each channel should be higher than the effective bandwidth of 480 kHz, and the minimum carrier frequency should be higher than 0.5EBD = 240 kHz. Assuming that *f*_1_ < *f*_2_ < *f*_3_ < *f*_4_:


$$\begin{array}{c} \left\{ \begin{array}{c} @l@f_{\text{1}}\geq 240kHz\\ f_{\text{2}}\geq f_{\text{1}}+480kHz\\ f_{\text{3}}\geq f_{\text{3}}+480kHz\\ f_{\text{4}}\geq f_{\text{3}}+480kHz \end{array}\right. \end{array}$$ (18)

In addition, for the noise M(t), the maximum frequency is *f*_4_-*f*_1_. To ensure that the noise term does not alias with each target signal:


$$\begin{array}{c} (f_{\text{4}}-f_{\text{1}})+480kHz\leq f_{\text{1}} \end{array}$$ (19)

According to the constraints of Eq. (18) and (19), in the 4-channel multiplexed sensor system, the minimum carrier frequencies should be selected as *f*_1_ = 1.92 MHz, *f*_2_ = 2.40 MHz, *f*_3_ = 2.88 MHz and *f*_4_ = 3.36 MHz. In this study, the laser sweep rate was set to γ =1.249 × 10^13^ Hz/s, the speed of light *c* = 3 × 10^8^ m/s, and *n* = 1.46. From Equation (16), the fiber length differences between each sensing optical path and the reference optical path should be *l*_1_ = 31.41m, *l*_2_ = 39.27m, *l*_3_ = 47.12m, *l*_4_ = 54.97m, respective*l*y. Under this carrier-frequency a*l*location, the maximum frequency components of the noise term *M*(t), which are generated by mutual interference among different sensing beams, is determined by the difference frequencies *f*_4_-*f*_1_. Therefore, the maximum noise frequency is 1.44MHz. According to Carson’s bandwidth theorem, the effective bandwidth of each target channel is *EBD* = 480 kHz, and the lower boundary of the first carrier channel is 1.68MHz. Obviously, all difference-frequency components contained in M(t) are located below the demodulation band of the lowest carrier channel. Therefore, the noise term *M*(t), does not overlap with the target sensing channels and can be suppressed through bandpass filtering.

## III. Results

**Fig 5:**
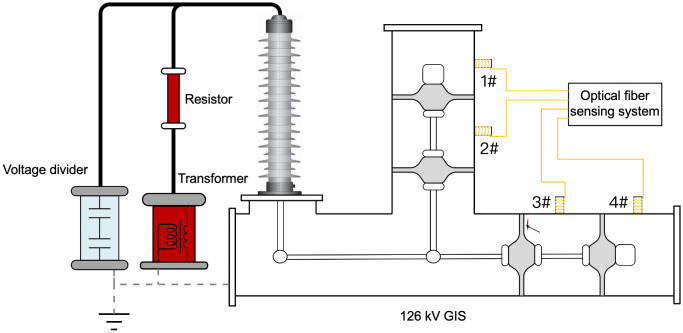
GIS metal particle defect detection experimental platform.

To understand the detection effect of the developed distributed optical fiber sensing system on the metal particle defects inside GIS equipment, an experimental detection platform was built based on a 126 kV GIS in this study, as shown in Fig 5.

**Fig 6:**
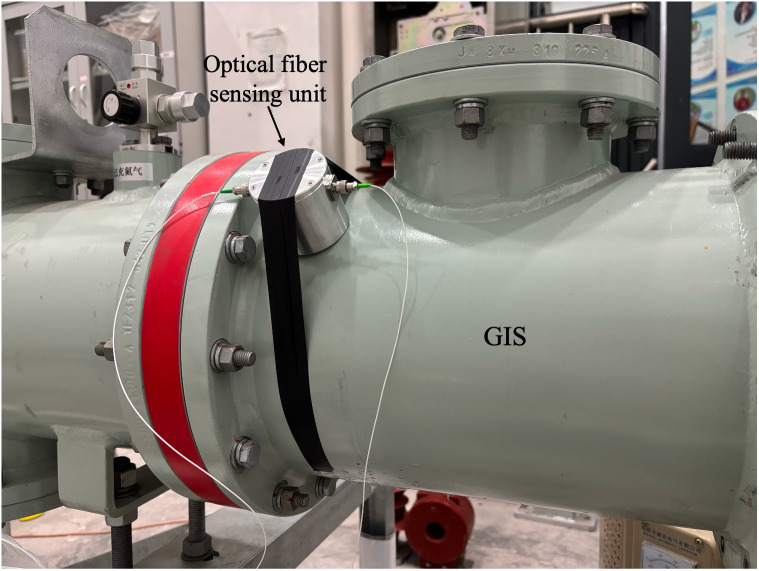
Actual picture of the sensing unit on the GIS shell.

The experimental GIS platform consisted of a high-voltage bushing and four test cavities, each separated by a pot-type spacer. The power frequency power supply applied voltage to the central guide rod in the cavity through the bushing. Two metal-particle defects were separately arranged and tested in different GIS cavitys. In the first experiment, a 10 mm aluminum wire was placed on the surface of a pot-type spacer to simulate an spacer-surface discharge defect. The fiber-optic sensing unit mounted on the outer wall of this cavity was denoted as sensor 3#. In the second experiment, after removing the first defect, another aluminum wire was placed at the bottom of the GIS enclosure to simulate a tip-discharge defect. The corresponding fiber-optic sensing unit mounted outside this cavity was denoted as sensor 4. For the tip-discharge defect experiment, a conventional PZT sensor was also installed on the same cavity for comparison with the proposed fiber-optic sensing system. In both experiments, each cavity was filled with SF6 gas at 0.5 MPa, and the four fiber-optic sensing units were fixed on the outer walls of the four test cavities to synchronously detect GIS metal-particle defects. The installation of the sensing unit on the surface of the GIS shell is illustrated in Fig 6.

**Fig 7:**
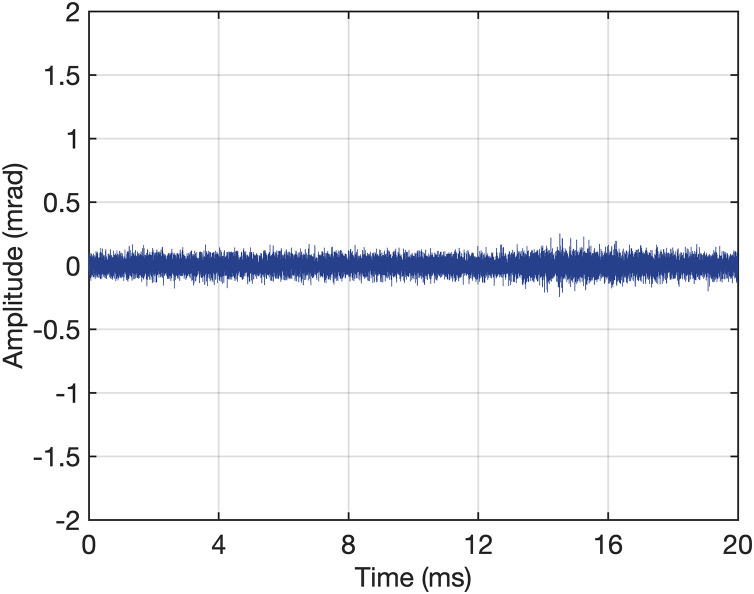
Demodulated signals detected by the sensor 1# under SF6 pressure of 0.5 MPa, applied voltage of 35 kV, and sampling rate of 1 MS/s.

**Fig 8:**
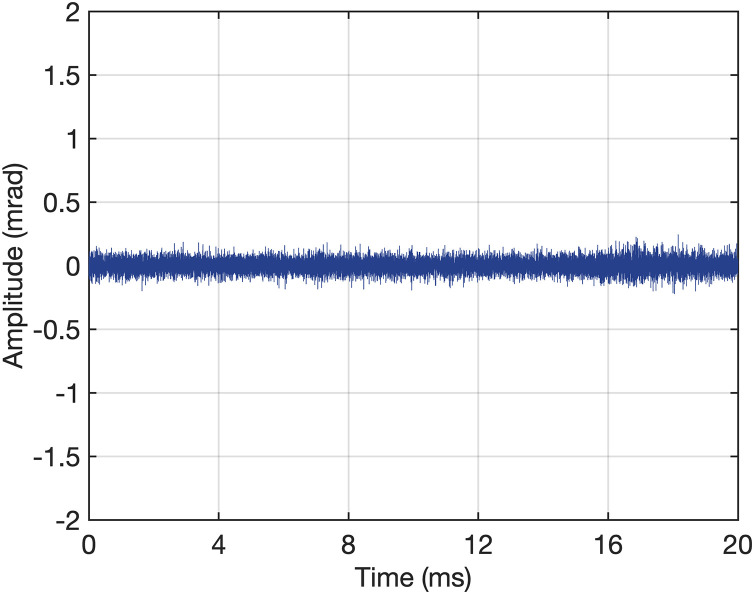
Demodulated signals detected by the sensor 2# under SF6 pressure of 0.5 MPa, applied voltage of 35 kV, and sampling rate of 1 MS/s.

**Fig 9:**
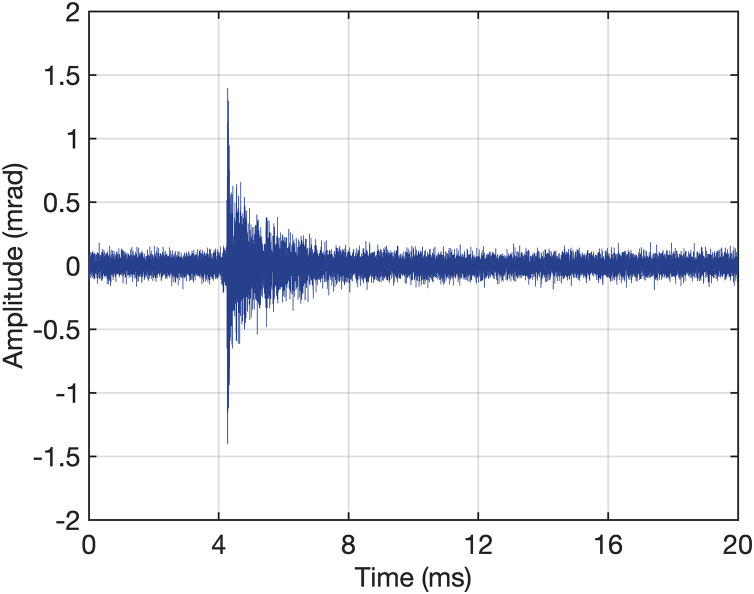
Demodulated signals detected by the sensor 3# under SF6 pressure of 0.5 MPa, applied voltage of 35 kV, and sampling rate of 1 MS/s.

**Fig 10:**
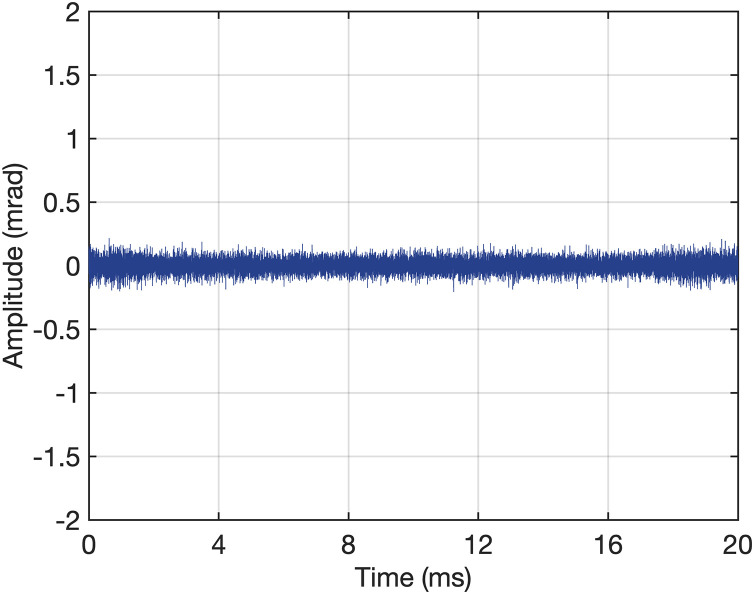
Demodulated signals detected by the sensor 4# under SF6 pressure of 0.5 MPa, applied voltage of 35 kV, and sampling rate of 1 MS/s.

For spacer-surface discharge defect, the experimental results are as follows. When the applied voltage was increased to 35 kV, the demodulation results of the signals synchronously detected by the four optical fiber sensing units within one power frequency cycle are shown in Figs 7–10.

The demodulation results show that only sensing unit 3 detected the pulsed acoustic signal generated by the metal-particle discharge, with a signal-to-noise ratio (SNR) of 21.7 dB, while the signals corresponding to the other sensor units were at the background noise level. Therefore, it can be concluded that a discharge defect exists in the cavity where sensing unit 3 is located, which is consistent with the cavity with defects preset before the experiment.

**Fig 11:**
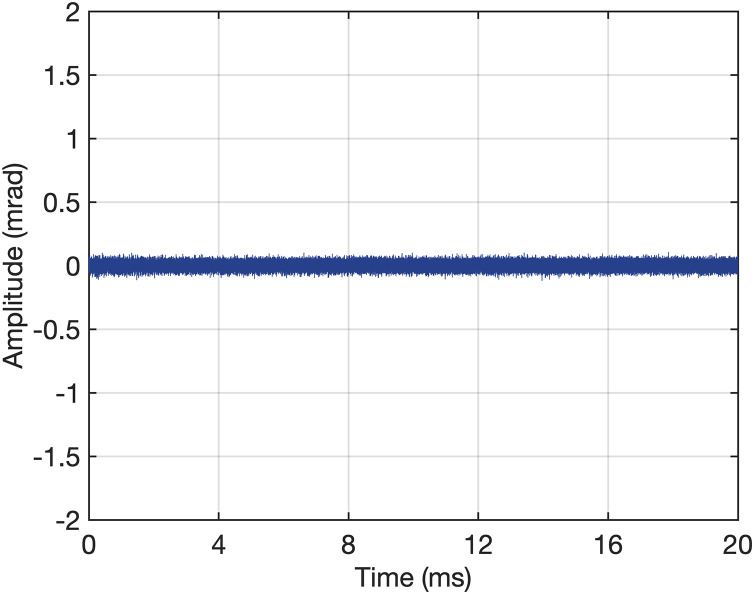
Demodulated signals detected by sensor 1# under SF6 pressure of 0.5 MPa, applied voltage of 46 kV, and sampling rate of 12.5 MS/s.

**Fig 12:**
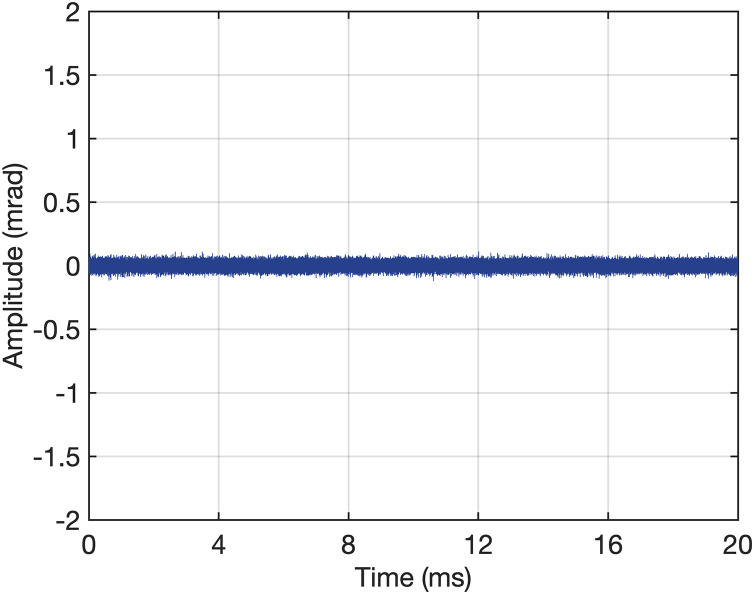
Demodulated signals detected by sensor 2# under SF6 pressure of 0.5 MPa, applied voltage of 46 kV, and sampling rate of 12.5 MS/s.

**Fig 13:**
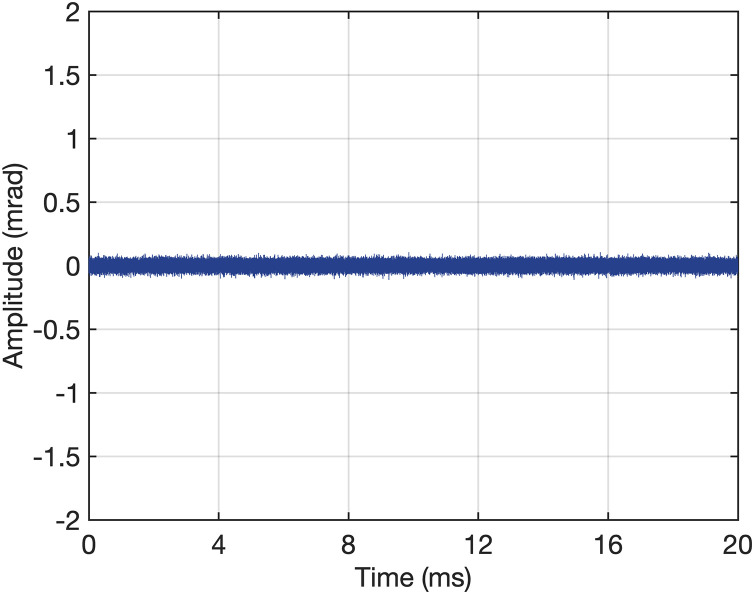
Demodulated signals detected by sensor 3# under SF6 pressure of 0.5 MPa, applied voltage of 46 kV, and sampling rate of 12.5 MS/s.

**Fig 14:**
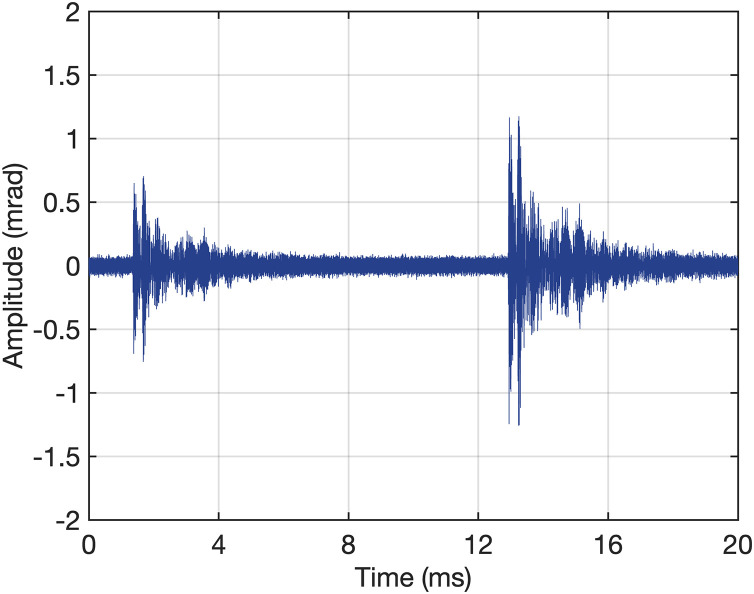
Demodulated signals detected by sensor 4# under SF6 pressure of 0.5 MPa, applied voltage of 46 kV, and sampling rate of 12.5 MS/s.

**Fig 15:**
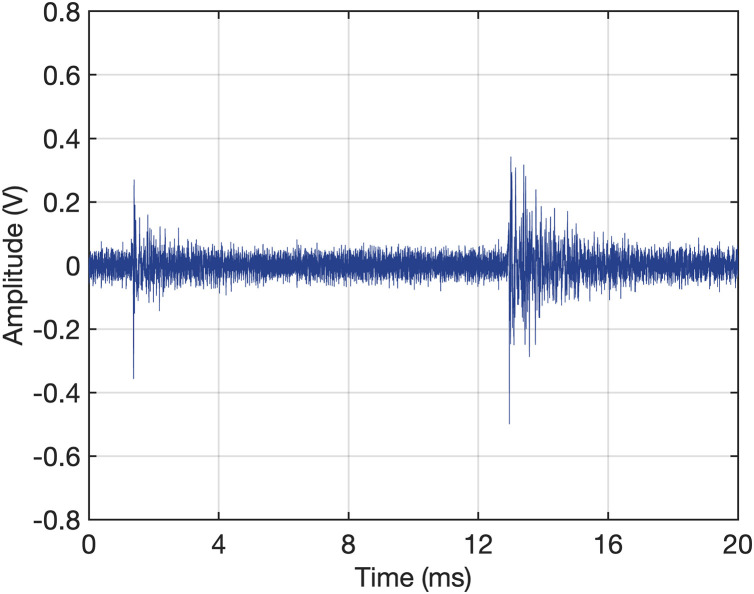
Signal detected by PZT sensor under SF6 pressure of 0.5 MPa, applied voltage of 46 kV, and sampling rate of 125 MS/s.

For tip-discharge defect, the experimental results are as follows. When the applied voltage was increased to 46 kV, the demodulation results of the signals synchronously detected by the four optical fiber sensing units within one power frequency cycle are shown in Figs 11–14. And the signal detected by PZT sensor is shown in Fig 15.

The demodulation results show that only sensing unit 4, which was mounted outside the cavity containing the defect, detected two pulsed acoustic signals within 0.02s, with SNRs of 19.4 dB and 23.5 dB, respectively. The signals detected by the other sensing units remained at the background-noise level. In addition, the PZT sensor also detected acoustic pulse signals. The detected pulses were temporally consistent with those obtained by sensor 4#. The SNRs of the PZT signals were approximately 16.9 dB and 19.8 dB, which were 2.5 dB and 3.7 dB lower than those of the fiber-optic sensing unit, respectively.

The above results demonstrate that the developed multiplexed fiber-optic sensing system can perform synchronous multipoint detection of GIS metal-particle defects using a common interrogation and demodulation system. In the two separately configured defect experiments, the insulator-surface discharge defect and the tip-discharge defect were correctly identified by sensing units 3 and 4, respectively, which were mounted outside the corresponding defect cavitys. The other sensing channels remained at the background-noise level, indicating effective channel separation. For the tip-discharge defect, the acoustic pulses detected by the fiber-optic sensing unit were temporally consistent with those detected by the PZT sensor installed on the same cavity, while the fiber-optic sensor showed higher SNRs. These results further verify the feasibility of the proposed method for cavity-level defect identification in GIS.

## IV. Conclusions

This study presents a distributed acoustic emission sensing method for GIS metal particle defect detection based on optical frequency-swept Michelson interferometry. By introducing frequency-dependent carriers through laser frequency sweeping, signals from multiple sensing units can be separated in the frequency domain, enabling frequency-division multiplexing with a single demodulation system.

From a system design perspective, the multiplexing performance is governed by the relationship between carrier spacing and signal bandwidth, and the difference-frequency noise term M(t). Based on Carson’s theorem, the effective bandwidth of each sensing channel is determined to be 480 kHz. To suppress inter-channel crosstalk and avoid aliasing with system noise, the carrier frequency interval must exceed this bandwidth, and the minimum carrier frequency must be higher than half of it. Accordingly, the optical path differences of the sensing fibers are quantitatively configured to ensure reliable channel separation, providing a practical design criterion for multi-channel distributed sensing systems.

Based on the proposed principle, a four-channel multiplexed fiber-optic sensing system was designed and experimentally tested on a 126 kV GIS platform. Two separately configured metal-particle defect scenarios were investigated, including an spacer-surface discharge defect and a tip-discharge defect. The experimental results show that the proposed system can detect discharge-induced AE signals generated by both defect types. The sensing units mounted outside the corresponding defect cavitys exhibited clear pulsed acoustic responses, with SNRs of 21.7 dB for the insulator-surface discharge defect and 19.4 dB/23.5 dB for the two acoustic pulses detected under the tip-discharge defect. In contrast, the other sensing channels remained at the background-noise level. These results demonstrate that the proposed method can identify the cavity containing the defect among the monitored cavities and verify the feasibility of frequency-swept multiplexed fiber-optic AE sensing for GIS metal-particle defect detection.

It should be noted that the present study demonstrates cavity-level defect identification rather than precise spatial localization within a GIS cavity. The experimental validation was conducted under laboratory conditions using two defect type and a four-channel sensing configuration. For applications requiring larger channel counts, the scalability of the proposed method should be further investigated. The carrier-frequency allocation must consider the effective bandwidth of each sensing channel, the available laser sweep rate, the bandwidth of the photodetector and data-acquisition system, and the cumulative difference-frequency noise term M(t). As the number of multiplexed channels increases, the number of mutual interference terms also increases, which may cause carrier-frequency crowding and increase the risk of crosstalk. Future work will focus on optimizing carrier-frequency allocation, increasing the number of multiplexed channels, suppressing channel crosstalk, and validating the system under more diverse defect types, defect locations, and field-like GIS operating conditions.

## Supporting information

S1 Data Raw numerical data underlying Figs 7, 8 and 9. ZIP archive containing CSV files of the raw data used for figure generation and statistical analyses.
(ZIP)
